# Functional connectivity differences in the default mode and frontoparietal networks in older adults with Down syndrome are related to obstructive sleep apnea

**DOI:** 10.1002/alz70862_110032

**Published:** 2025-12-23

**Authors:** Lisa M. Taylor, Jenna N. Adams, Natalie DiProspero, Liv McMillan, Mithra Sathishkumar, Eric Doran, Florence Lai, H. Diana Rosas, Adam Brickman, Elizabeth Head, Mark Mapstone, Nicole Schupf, Wayne Silverman, Ira T. Lott, Michael A. Yassa

**Affiliations:** ^1^ University of California, Irvine, Irvine, CA USA; ^2^ Harvard/Massachusetts General Hospital, Boston, MA USA; ^3^ Massachusetts General Hospital, Charlestown, MA USA; ^4^ Columbia University, New York, NY USA

## Abstract

**Background:**

There is disrupted functional connectivity (FC) of the default mode network (DMN) and frontoparietal network (FPN) in adults with obstructive sleep apnea (OSA). DMN FC is also particularly sensitive to disruption in Alzheimer’s disease (AD). However, there is limited research investigating if OSA affects FC in Down syndrome (DS), despite the prevalence of both OSA and AD pathology within this population. We examined if DMN and FPN FC are also disrupted in older adults with DS and OSA, and whether this is dependent on concurrent cognitive impairment due to AD.

**Method:**

Seventy‐five participants from the Alzheimer Biomarkers Consortium – Down syndrome (ABC‐DS) study (age 49.93+6.6) underwent resting‐state functional MRI. For our seed‐based connectivity approach, data were harmonized and processed using the CONN Toolbox with the default MNI preprocessing pipeline. FC strength was calculated (1) between regions‐of‐interest (ROIs) within each network (“within‐network FC”) and (2) between ROIs in each network and all other ROIs outside of the network (“between‐network FC”). Group differences in FC were assessed based on self‐reported OSA and consensus diagnosis of cognitive status – cognitively stable (CS) or cognitively impaired (CI; mild cognitive impairment or dementia).

**Result:**

There was a significant interaction between OSA and diagnosis for within‐ and between‐DMN FC (*p*‐values<0.05). The CS‐OSA group had higher FC than the CI‐OSA group. There was also a trend showing higher FC in CS‐OSA compared to CS‐nonOSA. For within‐ and between‐FPN FC, the same groups differences were observed, though the interaction was trending (*p*‐values<0.10). FC did not differ between CS and CI individuals without OSA, nor between CI groups with and without OSA.

**Conclusion:**

We observed higher within‐ and between‐DMN FC in the CS‐OSA compared to the CI‐OSA group; however, this difference was not observed between the CS‐nonOSA and CI‐nonOSA groups. This result suggests a unique FC signature for individuals with DS and OSA prior to AD‐related cognitive impairment. These findings suggest that there’s a unique FC signature for individuals with DS and OSA such that FC is initially elevated prior to AD‐related cognitive impairment then is reduced following AD symptom onset.

